# The association between temperament features and childhood traumas in patients with juvenile myoclonic epilepsy

**DOI:** 10.3906/sag-1912-18

**Published:** 2020-08-26

**Authors:** Alişan Burak YAŞAR, Ceyhun SAYMAN, Serap ERDOĞAN TAYCAN, Yılmaz ÇETİNKAYA, Anıl GÜNDÜZ, Hülya TİRELİ

**Affiliations:** 1 Department of Psychology. İstanbul Gelisim University, İstanbul Turkey; 2 Department of Norology, Haydarpaşa Numune Training and Research Hospital, İstanbul Turkey; 3 Department of Psychiatry, Haydarpaşa Numune Training and Research Hospital, İstanbul Turkey; 4 Department of Clinical Psychology, İstanbul Kent University, İstanbul Turkey

**Keywords:** Juvenile myoclonic epilepsy, temperament, childhood traumas, depression, anxiety

## Abstract

**Background/aim:**

Epilepsy is a common chronic neurological problem that impairs daily activities, functionality, and quality of life. Childhood traumas (CTs) are known to be critical factors in the onset or development of many psychiatric and medical disorders. They also play a critical role in the development of temperament and personality. This study aimed to investigate the association between CTs and common temperament patterns and features seen in epilepsy patients.

**Materials and methods:**

The study included 38 patients who were diagnosed with juvenile myoclonic epilepsy (JME) and volunteered to participate in the study. In addition to the sociodemographic form and questions on disease features, Structured Clinical Interview for DSM-IV Axis I Disorders, Temperament Evaluation of Memphis, Pisa, Paris and San Diego Questionnaire (TEMPS-A), Childhood Trauma Questionnaire (CTQ), Beck Depression Inventory (BDI), and Beck Anxiety Inventory (BAI) were administered to all participants. In the present study, a cut-off value of 35 was used for the CTQ scale. The patients with CTQ scores lower than 35 (50%, n = 19, Group 1) and the patients with CTQ scores above 35 (50%, n = 19, Group 2) were compared.

**Results:**

The comparison of TEMPS-A and its subscale scores in the JME patients in the groups with CTQ scores above or below a cut-off value detected significant differences between the groups in depressive and irritable temperament scores. The mean BDI scores were also different between the two groups. Furthermore, a significant positive correlation was detected between the disease duration, anxiety, and depression scores in the JME patients. A significant relationship was detected between the emotional neglect subscale score of the JME patients and the BDI scores. A significant positive correlation was found between the total disease duration, BDI, and BAI. Significant moderate-level relationships were found between the BDI score and irritable, depressive, cyclothymic, and anxious temperaments and between the BAI score and irritable, depressive, cyclothymic, and anxious temperaments.

**Conclusion:**

Several temperamental features of JME patients are related to CTs. More depressive symptoms are seen in JME patients with higher disease durations.

## 1. Introduction

Epilepsy is a common chronic neurological disease in which temperament features associated with the disease were defined [1]. Childhood traumas (CTs) and psychiatric comorbidities have been frequently investigated in epilepsy patients. These are accompanied by significant difficulties related to the course of disease and treatment. The most common comorbidity in epilepsy is major depressive disorder [2–4].

Juvenile Myoclonic Epilepsy (JME) is one of the subtypes of epilepsy [5]. JME constitutes 5–10% of all epileptic cases and 18% of idiopathic generalized epilepsies. The incidence of JME was 1/100,000 [6]. JME is one of the most common epilepsies in young adults. Its prevalence is so high that all generalized tonic-clonic seizures with adolescence onset should be accepted as JME until proved to be otherwise [7]. There is a trial about the onset, etiology, and clinical course of patients with JME; and several temperament features have been defined [8]. 

Genetic background and CTs were two factors defined in relation to temperament [9]. Many personalities and temperament features have been defined to be related with CT, especially those with B-cluster personality features [9–12]. Personality features in some epilepsy patients were termed ‘interictal behavior syndrome,’ ‘interictal personality syndrome,’ or ‘epileptic personality’ [13]. Effects of seizures or other long-term effects of interictal spike activity in EEG, head traumas caused by falls or crashes associated with recurrent seizures, and biological factors such as possible brain lesions and drug effects have been suspected in the etiology of personality changes observed in epilepsy. In addition, psychosocial factors such as stigmatization, low self-esteem, and social isolation are also believed to be among these factors [14]. A controlled study comparing temperament features of epilepsy patients and controls also found that irritable temperament was significantly more common in epilepsy patients compared with the control group [8]. It was suggested that sequelae of epileptic seizures adversely affects personality development, and may lead to the development of the features of personality disorder. Especially, irritable and dysthymic temperaments are commonly reported in epilepsy patients [15]. Akiskal and Mallye have described irritable temperament with such features as early-onset (<21 years of age), rare euthymic mood, widespread pessimism (irritability and getting angry easily), having a tendency to contemplate, being critical and complaining, making humorless jokes, forcing close relationships despite being unwanted, dysphoria, restlessness, and impulsiveness. They described subthreshold dysthymic temperament features as early-onset (<21 years of age), intermittent low-intensity depression, habitual long sleeping hours, anhedonia, and having a tendency to keep low energy for psychomotor activities, being pessimistic, hopeless, jolly, quiet, passive, undecided, skeptical, critical, complaining, being inclined to contemplate, being conscientious, self-disciplined, self-criticizing, self-punishing, and overthinking about failures, insufficiencies, and adversities [8,16,17].

Previous studies suggest that a part of psychiatric symptoms of epilepsy patients; in other words, “epileptic personality” may be explained by temperament features [18]. Epileptic personality was described with these terms: egocentric, viscous, combatant, and mystique [19]. Gastaut et al. observed elevated mood, viscosity, hypoactivity, and hyposexuality in epileptic patients [20]. Today, ‘epileptic personality’ is used both as a stigma, and also to define many emotional and behavioral symptoms developing in patients with epilepsy. However, these studies provide no data about past traumatic experiences.

The effects of traumatic experiences in the childhood period on personality development have been known for an extended period of time [21,22]. A history of childhood abuse or neglect has been reported in many patients with personality disorders [23,24]. In the DSM-IV, 10 out of the 12 personality disorder categories were associated with childhood abuse or neglect. Several types of childhood maltreatment were associated with specific personality disorder symptoms [23]. CT was found to be strongly associated with dysfunctional temperament features. Emotional abuse and neglect have been emphasized in a trial [23]. Dysfunctional temperament profiles were found to be associated particularly with emotional trauma and sexual abuse. Several studies found associations between several adaptive features with physical abuse and neglect, especially in males [25]. In a study evaluating nonepileptic patients, CT was found to be associated more commonly with cyclothymic and depressive temperaments [22].

The rate of CT is known to be high in epilepsy patients [26]. There may be a bilateral relationship between these two conditions. The presence of a chronic disease like epilepsy may cause children to be more prone to the adverse effects of trauma. In addition, the presence of a biological vulnerability may increase the risk of disease onset after the addition of traumatic experiences. In this context, it is noteworthy that our study sample included JME patients having a stronger neurobiological background. 

In light of these findings, evaluating the associations between common temperament features in epilepsy patients and the presence and severity of CTs may help to predict the prognosis and plan psychotherapeutic interventions. This study assessed the hypothesis that CTs were related to common temperament features in JME patients.

## 2. Materials and methods

### 2.1. Participants and sample selection

All of the 120 consecutive patients in the follow-up list of the Epilepsy Clinic of Haydarpaşa Numune Research and Training Hospital were called and informed about the study. Among them, 42 volunteers who could be reached and accepted to participate were involved in the study after signing the informed consent forms. Among these 42 patients, two were excluded because they were illiterate, and two were excluded because they had psychotic symptoms. The final analysis included 38 epileptic patients (29 females and 9 males) between 18 and 50 years of age. The study protocol was approved by the institutional review board at Haydarpaşa Numune Research and Training Hospital. All participants provided informed consent to be included in the study.

### 2.2. Sociodemographic and disease features form

This interview form was prepared by the researchers for this study; and it included questions about the patients’ medical and epileptic disease history. This form included items related to age, sex, marital status, occupation, level of education, place of residence, medical history, family history, the time of the last generalized seizure, the longest duration of years without a seizure, and drug groups used by the patients.

### 2.3. Structured Clinical Interview for DSM-IV Axis I Disorders (SCID-1)

SCID-1 is a semistructured clinical interview scale developed by First et al. to make Axis I diagnosis [27]. The validity and reliability study for the Turkish version was performed by Corapcioglu et al. [28].

### 2.4. Temperament Evaluation of Memphis, Pisa, Paris, San Diego Autoquestionnaire (TEMPS-A)

The original form of TEMPS-A was developed by Akiskal et al. in 1997, and it included 109 items for males and 110 items for females. On the other hand, the Turkish form includes 99 items. It is a self-report Likert-type scale that should be filled based on the whole life of a person. The items are answered as ‘true’ or ‘false.’ It contains five subscales as depressive, cyclothymic, hyperthymic, irritable, and anxious. The test-retest reliability of the subscales in the Turkish version was between 0.73 and 0.91, and Cronbach’s alpha coefficients were between 0.77 and 0.85 [29].

### 2.5. Beck Anxiety Inventory (BAI)

The BAI is a self-report scale developed by Beck to measure the severity of anxiety symptoms. It includes 21 items, each one scored from 0 to 3. The validity and reliability study of the Turkish version was performed by Ulusoy in 1998 [30].

### 2.6. Beck Depression Inventory (BDI)

The BDI was developed by Beck to measure somatic, emotional, cognitive, motivational, and psychomotor symptoms observed in depression. It is a self-report scale containing 21 questions, each of which is scored from 0 to 3 [31]. A score of 17 or above is considered clinically significant. The validity and reliability study of the Turkish version was performed by Hisli in 1988 [32].

### 2.7. Childhood Trauma Questionnaire (CTQ)

The CTQ was developed by Bernstein et al. in 1994, and it evaluated sexual, physical, and emotional abuse as well as emotional and physical neglect [33]. This Likert-type self-report scale includes 28 different items, which are scored from 1 to 5. The validity and reliability study of the Turkish version was performed by Sar et al. [34]. The subscale scores are between 5 and 24, and the total score is between 25 and 125. A score of ≥6 points is considered to be significant for sexual and physical abuse, ≥8 points is considered to be significant for physical neglect and emotional abuse, and ≥13 points is considered to be significant for emotional neglect. A total score of 35 is considered as a cut-off value. The patients with CTQ scores lower than 35 (50%, n = 19, Group 1) and the patients with CTQ scores above 35 (50%, n = 19, Group 2) were compared [35].

### 2.8. Location of the study

Epilepsy Clinic of the Department of Neurology, Haydarpaşa Numune Training and Research Hospital, Istanbul, Turkey.

### 2.9. Statistical evaluation

For data analyses, SPSS was used (SPSS for Windows version 16.0, SPSS Inc., Chicago, USA). Descriptive statistics were given as mean, standard deviation, frequency, and interquartile ranges. Independent samples *t* -test was used to compare quantitative data between the groups when the data were normally distributed, and the Mann–Whitney U test was used when the data did not have normal distribution. Spearman and Pearson tests were used to evaluate correlations. The results were evaluated with a 95% confidence interval and a significance level of P < 0.05. A cut-off value was used for the total scores from the CTQ. A correlation table was prepared to evaluate the correlations between the scores on the CTQ subscales, TEMPS-A subscales, BDI, and BAI. 

## 3. Results

### 3.1. Sociodemographic findings

Of the patients, 76.3% (n = 29) were females, and 23.7% (n = 9) were males; 57.9% (n = 22) were unemployed, and 42.1% (n = 16) were employed. The mean age was 29.7 ± 8.2 years. Level of education was at or below the primary school for 23.7% (n = 9), secondary school for 15.8% (n = 6), high school for 44.7% (n = 17), and university or above for 15.8% (n = 6). The parents had consanguineous marriages in 84.2% (n = 32). There was no family history of mental illness in 81.6% (n = 31) of the patients (Table 1).

**Table 1:** Sociodemographic characteristics (N = 38).

Characteristic		n (%)
Sex	Female	29 (76.3)
	Male	9 (23.7)
Occupation	Unemployed	22 (57.9)
	Employed	16 (42.1)
Education	Primary school or below	9 (23.7)
	Secondary school	6 (15.8)
	High school	17 (44.7)
	College or above	6 (15.8)
Consanguineous marriage	Absent	32 (84.2)
	Present	6 (15.8)
Family history of psychiatric disease	Absent	31 (81.6)
	Present	7 (18.4)

The mean age of diagnosis was 15.1 ± 5.3 years. The total duration of illness was between 2 and 33 years; the mean value was 14.2 ± 7.1 years. Three (7.9%) patients were not using any medications, 32 patients (84.2%) were using single antiepileptic, two patients (5.3%) were using two antiepileptic medications, and one (2.6%) patient was using three antiepileptic medications. A history of head trauma was present in 86.8% (n = 33). There was no cranial infection history in 97.3% (n = 37). There was no epileptic relative in the extended family of 63.1% (n = 24), and at least one epileptic relative was present in 36.8% (n = 14). Of the patients, 65.7% (n = 25) reported that the seizures were not triggered by stress, whereas 34.2% (n = 13) reported that the seizures were triggered by stress. The longest time without a seizure changed between 0 and 14 years, and the mean value was 4.7 ± 3.1 years.

### 3.2. Scale results, comparisons, and correlations

The mean values for TEMPS-A subscales were 5.63 ± 4.4 for anxious temperament, 5.95 ± 3.4 for depressive temperament, 11.16 ± 4.1 for hyperthymic temperament, 4.61 ± 3.6 for irritable temperament, and 7.45 ± 4.1 for cyclothymic temperament. Evaluation of TEMPS-A subscales using cut-off points to assess the dominant temperament revealed that one patient had anxious, two patients had depressive, one patient had hyperthymic, and one patient had irritable temperament. The mean scores for the subscales of CTQ were 6.13 ± 3.55 for sexual abuse, 10.58 ± 4.82 for emotional neglect, 6.61 ± 2.2 for emotional abuse, 7.34 ± 2.68 for physical neglect, and 5.82 ± 2.14 for physical abuse. The mean total score for CTQ was 36.47 ± 9.62. The mean BDI score of the patients was 11 ± 9.43, and the mean BAI score was 11.68 ± 11.88 (Table 2).

**Table 2:** TEMPS-A, CTQ, BDI, and BAI scores.

Scale		Mean ± SD
BDI		11 ± 9.43
BAI		11.68 ± 11.88
TEMPS-A	Anxious	5.63 ± 4.40
	Depressive	5.95 ± 3.40
	Hyperthymic	11.16 ± 4.10
	Irritable	4.61 ± 3.60
	Cyclothymic	7.45 ± 4.10
CTQ	Total score	36.47 ± 9.627
	Sexual abuse	6.13 ± 3.55
	Emotional neglect	10.58 ± 4.82
	Emotional abuse	6.61 ± 2.20
	Physical neglect	7.34 ± 2.68
	Physical abuse	5.82 ± 2.14

BAI: Beck Anxiety Inventory, BDI: Beck Depression Inventory, CTQ: Childhood Trauma Questionnaire, SD: Standard Deviation, TEMPS-A: Temperament Evaluation of Memphis, Pisa, Paris and San Diego Questionnaire

In the present study, a cut-off value of 35 was used for the CTQ scale [35]. The patients with CTQ scores lower than 35 (50%, n = 19, Group 1) and the patients with CTQ scores above 35 (50%, n = 19, Group 2) were compared. No difference was found between Groups 1 and 2 in the comparisons for the age of disease onset, disease duration, and the longest duration without the disease (Table 3). Groups 1 and 2 were also compared according to TEMPS-A subscale scores. Depressive temperament (P = 0.01) and irritable temperament (P *=* 0.04) scores were higher in Group 2. No significant difference was found in the BAI scores of the two groups (P *=* 0.62). The mean BDI score was relatively higher in Group 2 (Table 4).

**Table 3:** Comparison of the disease characteristics of the two groups.

Disease characteristic	Group 1 (CTQ < 35) n = 19	Group 2 (CTQ ≥ 35) n = 19	p
Age of disease onset (years)	21.11 ± 6.26	17.89 ± 4.14	0.37
Duration of disease (years)	13.37 ± 7.41*	15.21 ± 6.92*	0.43*
Longest duration without a GTCS (years)	19.24 ± 3.67	19.76 ± 2.53	0.88

Mann–Whitney U test *Independent groups t-test CTQ: Childhood Trauma Questionnaire, GTCS: Generalized Tonic-Clonic Seizures

**Table 4:** Comparison of TEMPS-A, BDI, and BAI scores of the two groups.

		Group 1 (CTQ < 35)n = 19	Group 2 (CTQ ≥ 35)N = 19	P
TEMPS-A	Anxious temperament	18.5 ± 4.16	20.9 ± 4.85	0.419
	Depressive temperament	15.3 ± 2.59	23.6 ± 3.75	0.019
	Hyperthymic temperament	17.4 ± 4.11*	21.5 ± 4.15*	0.247*
	Irritable temperament	15.9 ± 3.65	23 ± 3.47	0.046
	Cyclothymic temperament	16.20 ± 3.97	22.70 ± 3.87	0.067*
BDI		15.74 ± 9.67	23.26 ± 8.97	0.036
BAI		18.63 ± 13.05	20.37 ± 10.95	0.629

Mann–Whitney U test *Independent groups t-test BAI: Beck Anxiety Inventory, BDI: Beck Depression Inventory, CTQ: Childhood Trauma Questionnaire, TEMPS-A: Temperament Evaluation of Memphis, Pisa, Paris and San Diego Questionnaire

The correlations between the total duration of disease, age of disease onset, BDI score and BAI score were also evaluated. Significant and positive correlations were found between total duration of illness and BDI score (P *=* 0.049; *r* = 0.32) and BAI score (P *=* 0.048; *r* = 0.32).

Correlations between the CTQ and TEMPS-A subscales were evaluated. Moderate-level positive correlations were found between CTQ and irritable temperament (P *=* 0.017, *r* = 0.38) and depressive temperament (P *=* 0.01, *r* = 0.41). A positive and weak correlation was found between the physical abuse subscale of CTQ and hyperthymic temperament (P *=* 0.04, *r* = 0.32). The other correlations between CTQ subscales and temperament were not significant (P > 0.05) (Table 5).

**Table 5:** Correlations between CTQ scores and TEMPS-A scores.

	TEMPS-A									
	Irritable		Depressive		Cyclothymic		Hyperthymic		Anxious	
CTQ	r	P	r	P	r	P	r	P	r	P
Emotional abuse	0.385	0.017	0.415	0.01	0.27	0.101	–0.037	0.825	0.114	0.497
Physical abuse	0.224*	0.175	0.282*	0.087	0.156	0.351	0.327*	0.045	–0.04	0.809
Physical neglect	–0.037	0.824	–0.209	0.207	–0.101	0.548	–0.297	0.071	0.049	0.77
Emotional neglect	0.212	0.201	0.22	0.185	0.306*	0.062	0.009*	0.958	0.108	0.517
Sexual abuse	0.226	0.173	0.229	0.166	0.253	0.125	0.194	0.242	0.105	0.53
Total	0.327*	0.045	0.282	0.087	0.304	0.063	0.001	0.994	0.133	0.427

Spearman’s correlation analysis *Pearson’s correlation analysis CTQ: Childhood Trauma Questionnaire,, TEMPS-A: Temperament Evaluation of Memphis, Pisa, Paris and San Diego Questionnaire

The correlations between CTQ subscales and BDI and BAI were also evaluated. Significant positive correlations were found between BDI score and CTQ total score (P *=* 0.01; *r* = 0.40) and CTQ emotional neglect subscale score (P *=* 0.04; *r* = 0.32). 

The correlations between TEMPS-A subscale scores and BAI and BDI scores were also evaluated. Moderate-level significant correlations were found between BDI and irritable temperament (P < 0.01; *r* = 0.65), depressive temperament (P < 0.01; *r* = 0.52), cyclothymic temperament (P < 0.01; *r* = 0.54), and anxious temperament (P < 0.01; *r* = 0.58). There were moderate-level significant correlations between BAI and irritable temperament (P = 0.02; *r* = 0.48), depressive temperament (P < 0.01; *r* = 0.46), cyclothymic temperament (P < 0.01; *r* = 0.50), and anxious temperament (P < 0.01; *r* = 0.58).

## 4. Discussion

The effects of traumatic childhood experiences on personality development have long been known [23,24]. This study aimed to evaluate the effects of CTQ on temperament characteristics in JME patients and also its relationships with anxiety and depression scores. Epileptic individuals might have experienced much more CT than healthy controls, and they may receive a diagnosis of posttraumatic stress disorder [36].

Personality features observed in JME were reported to be associated with the onset of disease at adolescence, diagnosis at adolescence, and the requirement for continuous treatment. Cluster B personality disorders (being histrionic, passive-aggressive, and having borderline), which are related to impulsive behavior, are more common in JME [37,38]. A study evaluating 170 JME patients revealed that moderate personality disorders were the most common finding, and this could be a part of the clinical picture [39]. Interestingly, CT was not evaluated in those studies. Traumatic experiences are known to impair emotion regulation [40].

Personality disorders proposed in JME are different from focal epilepsies. In those patients, personality features such as loss of attention, impulsiveness, suggestibility, impatience, variable self-esteem, to be undisciplined, and emotional fluctuation might be seen [36]. Previous studies suggested frontal lobe dysfunction in JME, especially in the prefrontal cortex. Functional and structural frontal lobe abnormalities have been detected. Diffuse neural pathway abnormalities were suggested in the thalamus, hippocampus, and corpus callosum in addition to the frontal lobe. Problems were reported in dimensions such as executive functions and attention [41]. Personality features in JME may be due to the onset of disease at adolescence, the patients being diagnosed at the adolescence period, and problems due to continuous treatment requirements. Cluster B personality disorders (being histrionic, passive-aggressive, and having borderline), which are related to impulsive behavior, are more common in JME [37,38]. In this context, investigation of a group that is relatively more homogenous and whose neurobiology has been investigated more thoroughly is strength of our study.

In psychiatric patients with a history of abuse, the incidence of clinically significant EEG abnormalities (spike waves, sharp waves, and paroxysmal slowing) was more common than children without psychiatric diseases [42]. Abnormal EEG findings were detected in 72% of children with a history of physical or sexual abuse [43]. Patients who reported that stress changed their seizure frequency were found to have more frequent CT, especially emotional abuse, compared with those who did not report a stress-related change in seizure frequency [44]. In a study that screened temperament features in epilepsy it was revealed that epilepsy patients had higher scores in all affective temperaments except hyperthymic temperament. In the present study, significant relationships were found among depressive temperament, cyclothymic temperament, irritable temperament, and anxious temperament [45]. In several studies, anxious and irritable temperaments were found to be higher in epileptic patients, and anxious temperament was found to be related to major depressive disorder. A tendency for affective temperament was emphasized in epilepsy patients [45]. In our study, all of the anxious, irritable, depressive, and cyclothymic temperaments were found to be associated with anxiety and depression scores. An association was not found only for hyperthymic temperament, which is consistent with the temperament study mentioned above. A significant association with anxiety features, in addition to depressive features, suggests that all of these temperament features are associated with depression and anxiety symptoms, although it does not have diagnostic significance. In another study, it was emphasized that irritable temperament in epilepsy patients was associated with depressive temperament [8]. 

A commonly used cut-off value for CTQ total score is 35 [35]. In our study, the patients were classified as Group 1 or Group 2 based on the CTQ score being above or below the cut-off value. A comparison of the TEMPS-A scores of these groups indicated differences in the depressive and irritable temperament scores. In patients with CTQ scores higher than 35, depressive and irritable temperament scores were significantly higher. There was a difference between epilepsy patients and normal subjects between 4 temperament features including anxious and cyclothymic temperaments. Surprisingly, in our study only 2 of them were associated with trauma. The impact sizes of genetic factors and stress, which are known to have roles in the development of temperament, may be the subject of future studies. In this multifactorial process, these factors may have variable roles in different temperaments.

Evaluation of the subscale scores in our study demonstrated significant but weak negative correlations between the physical abuse subscale of CTQ and hyperthymic temperament, which is consistent with the findings in previous studies on CTs and temperament, excluding epilepsy [25]. In our study, there were positive correlations between the emotional abuse subscale of CTQ and irritable temperament and depressive temperament. This finding is also consistent with the general CT findings [25].

Features of irritable temperament, which is related to epileptic patients and especially with early-onset JME patients, might be associated with emotional neglect that might be facilitated by the disease or the burden of this disease on the family. The presence of early-onset JME and its burden on the family system in our patient sample could be considered a traumatic experience, and this might have contributed to the development of temperament. It could be hypothesized that traumatic life experiences in the presence of a biological tendency contribute to the development of both temperamental features and epilepsy. Therefore, an epileptic person may have the risk of abuse-neglect at the same time. It is known that caring for an epileptic child is a significant burden for the family both physically and mentally when the seizures are resistant to treatment and when multiple medications are required [46]. Early disease onset, overprotective family, psychosocial factors related to the disease, and stigmatization might be related to impaired quality of life [47]. This relationship may augment the effect of CT on an individual or may even decrease the resilience of an individual against the effects of stress. Accordingly, one of the causes of epileptic temperament may be “CTs associated with epilepsy” [47]. Considering that irritable temperament is commonly found in studies investigating temperament features in the epileptic patient group [8,45], this association in our study may be due to the overall frequency of irritable temperament features. 

In the comparison of BDI and BAI scores of the two groups that were classified according to the CTQ scores being above or below cut-off levels, a significant difference was detected only in BDI scores. Detection of more frequent depressive temperament in these patients may explain this relationship. Increasing depressive features with higher CTQ scores is consistent with the findings from nonepileptic populations [48]. Previous studies have shown that psychopathology and social dysfunction may determine the course of disease and comorbidities; and evaluations should be made regarding therapeutic interventions. For example, a clear relationship was found between the current frequency of seizures and anxiety levels, depression levels, perceived effects of epilepsy, perceived stigmatization scores, marital status, and employment status. At this stage, it will be helpful to emphasize that JME is an early-onset epilepsy subtype [49]. The mean age of onset in our study was 15 ± 5.3 years. Previous studies have reported that epilepsy has a significant psychosocial effect during the adolescence period, and these individuals define adolescence period as a difficult period in their lives [50]. An association between the duration of disease and depressive symptoms was also found in our study. 

According to all of these data, several factors might be considered for temperament features in early-onset JME patients and other epilepsies. Some of these factors may be biological, and others may be psychosocial. Detecting psychosocial factors is very important for both psychotherapeutic interventions and the quality of life of patients. Knowing temperament features and traumatic experiences of the patients are essential to develop psychotherapeutic interventions.

In conclusion, this study evaluated the effects of CT on temperament features and anxiety and depression scores in JME patients and revealed that irritable temperament and depressive temperament are significantly related to CTs in JME patients.
